# Chicken-derived RSPO1 and WNT3 contribute to maintaining longevity of chicken intestinal organoid cultures

**DOI:** 10.1038/s41598-022-14875-7

**Published:** 2022-06-22

**Authors:** Miriam J. Oost, Adil Ijaz, Daphne A. van Haarlem, Kitty van Summeren, Francisca C. Velkers, Aletta D. Kraneveld, Koen Venema, Christine A. Jansen, Raymond H. H. Pieters, Jean Paul ten Klooster

**Affiliations:** 1 Centre for Healthy Eating and Food Innovation, Faculty of Science and Engineering, Maastricht University, Campus Venlo, Venlo, The Netherlands; 2 Division Infectious Diseases and Immunology, Department Biomolecular Health Sciences, Faculty of Veterinary Medicine, Utrecht University, Utrecht, The Netherlands; 3 Innovative Testing in Life Sciences and Chemistry, Research Centre Healthy and Sustainable Living, University of Applied Sciences Utrecht, Utrecht, The Netherlands; 4 Division Farm Animal Health, Department Population Health Sciences, Faculty of Veterinary Medicine, Utrecht University, Utrecht, The Netherlands; 5 Division of Pharmacology, Utrecht Institute for Pharmaceutical Sciences, Faculty of Science, Utrecht University, Utrecht, The Netherlands; 6 Department of Animal Sciences, Cell Biology and Immunology Group, Wageningen University & Research, Wageningen, The Netherlands; 7 Department of Population Health Sciences, Institute for Risk Assessment Sciences, Utrecht University, Utrecht, The Netherlands

**Keywords:** Cell biology, Cell growth

## Abstract

Intestinal organoids are advanced cellular models, which are widely used in mammalian studies to mimic and study in vivo intestinal function and host–pathogen interactions. Growth factors WNT3 and RSPO1 are crucial for the growth of intestinal organoids. Chicken intestinal organoids are currently cultured with mammalian Wnt3a and Rspo1, however, maintaining their longevity has shown to be challenging. Based on the limited homology between mammalian and avian RSPO1, we expect that chicken-derived factors are required for the organoid cultures. Isolated crypts from embryonic tissue of laying hens were growing in the presence of chicken WNT3 and RSPO1, whereas growth in the presence of mammalian Wnt3a and Rspo1 was limited. Moreover, the growth was increased by using Prostaglandin E2 (PGE_2_) and a Forkhead box O1-inhibitor (FOXO1-inhibitor), allowing to culture these organoids for 15 passages. Furthermore, stem cells maintained their ability to differentiate into goblets, enterocytes and enteroendocrine cells in 2D structures. Overall, we show that chicken intestinal organoids can be cultured for multiple passages using chicken-derived WNT3 and RSPO1, PGE_2_, and FOXO1-inhibitor.

## Introduction

Poultry meat and eggs are low in production costs, are accepted in many cultures, and have a relatively low environmental impact compared to other sources of animal protein, because they produce significantly lower greenhouse gas emissions^1^. This makes it an important and efficient source of animal protein and essential for feeding the growing world population^2^. However, infectious disease control is a major challenge in poultry farming and antimicrobial drugs are often used to reduce the impact of intestinal infections. To reduce the contribution to risks of antimicrobial resistance to public health, strict regulations on antimicrobial use apply nowadays for livestock farming in the European Union^3^. However, there is still a need to reduce and prevent intestinal diseases, which has led to a continuous search for alternatives with e.g. antimicrobial, anti-inflammatory, antioxidant, or immune-fitness promoting properties^4,5^.

The gastrointestinal system plays a pivotal role in health, metabolism, immunity, and production performance in chickens. There is, for instance, a close interaction between the commensal gut microbiota and the host immunity, contributing to an efficient innate immune response to potential pathogens^6,7^. With the intestine playing an important role in chicken's health, strategies to promote or maintain intestinal health are warranted. For example, feed additives, such as mannan-oligosaccharide, have been used to stimulate intestinal immune responsiveness^8,9^. Most studies on feed additives, that promote intestinal health, were performed in vivo and require the use of experimental animals. Although these results are highly relevant to the field, in vitro strategies to study intestinal health are needed in view of in-depth mechanistic research and in the context of replacing, reducing and refining (the 3R’s) animal experimentation. Therefore, it is of great interest to have a near-physiological in vitro model to study direct host-microbe interactions and the effects of food components on the epithelial cell barrier of the intestine.

Organoids are advanced cellular models, which have been widely used in mammalian studies since first introduced by Sato and colleagues^10^. These self-organizing three-dimensional (3D) tissue cultures, derived from stem cells, represent the physiology of in vivo situations better than monolayer cell lines because stem cells can differentiate into different epithelial cell types present in the intestine, e.g. enterocytes, goblet cells, enteroendocrine cells, and Paneth cells. Most 3D organoids have the apical site of the epithelium at the inside of the 3D structure, which makes it more difficult to access. By additionally growing intestinal organoids in a two-dimensional (2D) way, studies concerning apical stimulation, gut hormone secretion, and mucus production can be done, as well as absorption studies and co-culture studies with immune cells and bacteria^11^.

For poultry, a limited number of studies on intestinal organoids have been reported^12–17^. So far, most of the studies did not show growth of more than one passage, however, for our research we require cultures that maintain their stem cells and thus have longevity, resulting in multiple passaging of these chicken intestinal organoids. Extrapolating from mammalian intestinal organoids, it is suggested that Matrigel and the growth factors Wnt3 and Rspo1 are crucial for the formation, development, and maintenance of organoid cultures through activation of the WNT-pathway^10^, which can be enhanced by the combination of a Glycogen synthase kinase-3β-inhibitor (CHIR99021) and a histone deacetylase inhibitor (valproic acid). Mammalian Wnt3 and Rspo1 have currently been used for chicken intestinal organoids, despite the difference in protein homology of these factors between chicken and mammalian origin. We expected that using mammalian-derived growth factors does not contribute to longevity of these cultures, and instead, require chicken-derived factors. Moreover, additional growth factors may be needed. Pierzchalska and colleagues showed that prostaglandin E2 (PGE_2_) has a positive effect on the growth of chicken embryo intestinal organoids^18,19^. Another factor that seems promising is the Forkhead box O1 (FOXO1) inhibitor AS1842856, because of its crosstalk with LGR5 signaling^20^. FOXO1 suppresses the WNT/β-catenin pathway and is known to regulate maintenance of stemness in multiple systems, including the intestine^21^. In this study, we show that chicken intestinal organoids can be cultured for multiple passages when using chicken-derived WNT3 and RSPO1, PGE_2_ and a FOXO1-inhibitor.

## Material and methods

### Medium and reagents

Components of the media formulations for different organoid experiments used in the study are listed in Supplementary Table 1, and details can be found underneath.

#### Basic culture medium (BCM)

Dulbecco's Modified Eagle Medium/Ham's F-12 (DMEM/F12) supplemented with 10% Fetal Calf Serum (FCS), 1% GlutaMAX, 1 mM sodium pyruvate, MEM Non-Essential Amino Acids, penicillin–streptomycin 10,000 U/mL (all Gibco, Life Technologies Limited, Paisley, UK).

#### Intermediate culture medium (ICM)

Basic Culture medium, supplemented with CHIR99021 4.3 µM (Sigma-Aldrich, Saint Louis, MO, USA, SML 1046), Y27632, a selective ROCK1 inhibitor, 10 µM (Absource Diagnostics GmbH, München, Germany), valproic acid 0.78 mM (Sigma-Aldrich, Saint Louis, MO, USA, P6273), DMH-1, a selective inhibitor of activin receptor-like kinase 2 ALK2), 1.3 µM (Sigma-Aldrich).

#### Chicken organoid culture medium (COCM)

Intermediate culture medium supplemented with FOXO1-inhibitor, AS1842856, 0.2 µM (Sigma-Aldrich), PGE_2_ 7.09 µM (Stemcell Technologies, Vancouver, Canada), chicken-specific WNT3 (1:5, in-house production; see below), and RSPO1 (1:5, in-house production).

#### Mouse organoid culture *medium (MOCM)*

Basic culture medium, supplemented with 1:5 of supernatant of Rspo1 expressing Hek293t cells (Sigma-Aldrich; SCC111), 1:5 of supernatant of L Wnt-3A cells (ATCC, Molsheim, France; CRL-2647), and the BMP4 inhibitor DMH1 1.3 µM (Sigma-Aldrich; D8946).

### In house production of chicken-specific growth factors WNT3/RSPO1

To produce chicken-specific growth factors WNT3 and RSPO1, we subcloned Chicken RSPO1-DYK (Genscript; OGa18707) and WNT3-DYK (Genscript; OGa25549D) cDNA clones into Tol2-containing E4 plasmid (Kind gift from Stefan Schulte-Merker^22^). Tol2 sequences allowed us to have stable genomic integration of RSPO1 and WNT3 cDNA in Hek293t cells upon Puromycin selection (Invitrogen; ant-pr-1). After selection, Hek293t -RSPO1 and -WNT3 were expanded in culture medium and when cells were 100% confluent for 2 days, supernatant was collected and centrifuged for 5 min at 295 × g. For organoid cultures, these supernatants were diluted 5 times in ICM.

### Protein alignments

The protein sequences of WNT3 and RSPO1 of chicken and its orthologues of mouse and human were found using NCBI BLAST (blast.ncbi.nlm.nih.gov/Blast.cgi). The following protein sequences were obtained from NCBI and used to make alignments: WNT3: NP_00107565.1 (Gallus gallus), NP_033547.1 (Mus musculus), NP_110380.1 (Homo sapiens). RSPO1: NP_001305373.1 (Gallus gallus), NP_001033722.1(Mus musculus), NP_619624.2 (Homo sapiens).

### Isolation of avian intestinal crypts and development of 3D intestinal organoids

Embryonic day 18 (ED18) Lohmann Brown chicken eggs (*Gallus gallus)* were obtained from the Department of Farm Animal Health, Faculty of Veterinary Medicine, Utrecht University, the Netherlands. For each isolation, both small and large intestines were collected and pooled from three chicken embryos *post-mortem*, and repeated three times. This method was conducted in accordance with protocols approved by the Department of Biomolecular Health Sciences, Division of Infectious Diseases & Immunology of the Utrecht University. Although according to European legislation for the protection of animals used for scientific purposes (Directive 2010/63/EU) experiments with embryonated chicken eggs are not considered animal experiments. The study was carried out in compliance with the ARRIVE guidelines.

**Figure 1:**
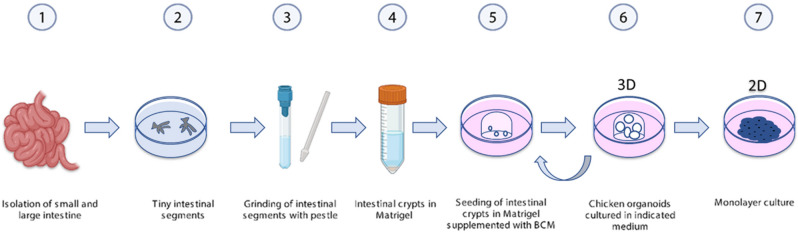
Schematic overview of the isolation and culture conditions of chicken intestinal organoid. (1) Isolation of small and large intestines (2) Intestinal tissue was cut into tiny segments and washed with PBS-EDTA. (3) To obtain a homogenous suspension, segments were minced using a Potter–Elvehjem PTFE pestle and glass tube. (4) After multiple centrifugation steps, the pellet was resuspended in ice-cold Matrigel (5) and seeded in a culture plate (6)**.** After polymerization of Matrigel, crypts were cultured with indicated media as defined in supplementary Table 1. (7) Monolayer cultures were obtained from three-dimensional cultures.

Immediately after collection, the intestines were placed in a petri dish containing 10 mL of 5 mM ice-cold phosphate-buffered saline-ethylenediaminetetraacetic acid (PBS-EDTA; Lonza, Basel, Switzerland). After washing with PBS-EDTA, the intestines were cut into small segments and placed into a 50 mL conical tube containing PBS-EDTA. To get a homogenous suspension of intestinal crypts, 2 mL of PBS-EDTA containing intestinal segments was minced using a potter–elvehjem PTFE pestle and glass tube (Sigma-Aldrich). The intestinal crypts’ suspension was collected in a 15 ml tube and centrifuged at 295×*g* for 5 min at 4 °C. After centrifugation, the supernatant was discarded and crypt aggregates were resuspended in 10 mL PBS-EDTA and centrifuged again. This was repeated up to 5 times until supernatants free of floating particles. Next, the crypts’ suspension was filtered through a 100-µm cell strainer (Corning, Amsterdam, the Netherlands) to get a uniform intestinal crypt solution. The filtrate was centrifuged at 295×*g* for 5 min at 4 °C and supernatant was discarded. The pellet was resuspended in 800 µL of ice-cold Matrigel (Corning; 354234). Two drops of 25 µL intestinal crypts-matrigel suspension, containing 25–50 crypts, were added per well in a pre-warmed 12-well culture plate (Corning). Following polymerization of Matrigel, 2 mL of organoid media (as indicated) was added per well and the culture plate was placed at 41 °C, 5% CO_2_, which is the optimal temperature to culture chicken cells, to mimic the body temperature of the chicken (Fig. 1). 3D intestinal organoids were passaged twice a week in a 1:4 ratio, by dissociating the organoids embedded in Matrigel drops in 1 mL fresh BCM through vigorous pipetting. After dissociation, the organoid solution was centrifuged at 295×*g* for 5 min at 4 °C. Supernatant was discarded and the organoids pellet was suspended in ice-cold Matrigel for seeding to expand the 3D intestinal organoids culture.

### Isolation of mouse intestinal crypts

The mouse intestinal crypts were isolated from the ileum as reported previously^23^. A female mouse C57/BL6, 6 months of age was sacrificed using carbon dioxide. The use of mouse intestine was approved by the local committee for care and use of animals at Utrecht University and all experiments were performed in accordance with the relevant guidelines and regulations. Only one surplus mouse was used for this study. The study was carried in compliance with the ARRIVE guidelines.

Villi were removed from the ileum, by scraping with a scalpel and the tissue was cut into small pieces, transferred to a tube with ice-cold PBS and washed three times with PBS (spun down at 295×*g* for 5 min between every wash step). Crypts that were isolated from the villi were resuspended in Matrigel, containing 25–50 crypts/25 µL, and two drops of 25 µL suspension were added to each well. The organoids were kept in ICM for 3 days, and medium was subsequently replaced with MOCM to stimulate organoid formation. These organoids were passaged once a week in a 1:4 ratio, containing 25–50 organoids/well, by dissociation of the organoids embedded in Matrigel drops in 1 ml fresh BCM through vigorous pipetting. After dissociation, the organoid solution was centrifuged at 335×*g* for 5 min at 4 °C. Supernatant was discarded and the organoids pellet was resuspended in ice-cold Matrigel for seeding to expand the 3D intestinal organoid culture.

### Conversion of 3D intestinal organoids to monolayer culture

To obtain 2D intestinal organoids, 3D intestinal organoids were used. Before seeding the monolayer chicken intestinal organoids, 12 mm sterile glass coverslips (Waldemar Knittel Glasbearbeitungs GmbH, Brunswick, Germany) were coated with Matrigel. This was done by placing the coverslips in a 24 well culture plate, and 40 µL of 2.5% ice-cold Matrigel solution in Dulbecco’s Phosphate Buffered Saline with calcium and magnesium (DPBS^+/+^) was added per well, followed by a 2 h incubation at room temperature (RT). The Matrigel solution was removed and the plate was air-dried for 20 min at RT.

To obtain a single-cell suspension, four wells of 3D intestinal organoids embedded in Matrigel were used to form one well of the monolayer organoids. The 3D intestinal organoids were dissociated mechanically by vigorous pipetting using a 1000-µL pipet in cold BCM. After dissociation, the solution was centrifuged at 131×*g*, 3 min at 4 °C. Supernatant was discarded and the pellet was resuspended in 200 µL 0.25% trypsin–EDTA (Gibco; 25200056) and incubated for 5 min at 37 °C. These steps were repeated until a single cell suspension was obtained (in general this required two steps), which was evaluated under a light microscope. The pellet containing the single-cell suspension was resuspended in 100 µL/well COCM and seeded on the matrigel-covered glass coverslips and an additional 600 µL of COCM was added per well and, subsequently, the culture plate was placed at 41 °C, 5% CO_2_. Intestinal organoid monolayers that were formed on glass coverslips reached almost 70% coverage within 2 days.

### Immunohistochemical staining

To identify various chicken intestinal epithelial cell lineages; glass coverslips containing the monolayer cultures were fixed with 200 µL of DPBS^+/+^ and 4% paraformaldehyde (PFA) solution for 1 h at RT. Later on, monolayer cultures were washed with 1 mL of DPBS^−/−^ and 10 mM glycine (Merck Millipore, Burlington, MA, USA) solution to quench the fixative. Following fixation, the monolayer cultures were blocked in 300 µL of blocking buffer consisting of DPBS^−/−^, 0.05% Tween-20 (Sigma-Aldrich), and 2% bovine serum albumin (Sigma-Aldrich) for 2 h at 4 °C. Coverslips were then stained with monolayer faced down on parafilm containing 25 µL of blocking buffer containing primary antibodies, as mentioned in Table 1, overnight at 4 °C. The next day, monolayer cultures were stained with secondary antibodies as mentioned in Table 1, for 1 h at RT protected from light. In addition, nuclear staining was performed by incubating the glass coverslips for 5 min in 300 µL blocking buffer containing 10 µg/mL 4,6-diamidino-2-phenylindole (DAPI) (Sigma-Aldrich) at RT. The samples were washed three times with 1 mL washing buffer containing DPBS^−/−^, and 0.05% Tween-20 in between the staining steps. The last washing step after nuclear staining was done with 1 mL distilled water and then the samples were mounted on Polysine® microscope slides (Menzel Glaser, Braunschweig, Germany) containing FluorSave mounting reagent (Merck Millipore).

**Table 1:** Characteristic of the used antibodies.

Antibody	Concentration	Species cross-reactivity	Target site	Host/Isotype
Anti-Sox9 AB5535 Sigma-Aldrich	1:500	Human, Mouse, Rat, Chicken	Sox9, a transcription factor and recognized marker for stem/progenitor cells	Rabbit
Anti-chromogranin-A Immunostar 20086	1:500	Buffalo, Chicken, Cow, Dolphin, Human, Mouse	Enteroendocrine cells	Rabbit
Anti-occludin Thermofisher 71-1500	1:100	Baboon, Bacteria, Chicken, Dog, Human, Mouse, Rabbit, Rat	Tight junction membrane	Rabbit
Donkey anti-Rabbit IgG (H + L) Highly Cross-Adsorbed Secondary Antibody, Alexa Fluor 488	1:1000	Rabbit	Gamma Immunoglobins Heavy and Light chains	Donkey/IgG

To determine the cross-reactivity of primary antibodies we performed staining of cryosections of embryonic day 18 (ED18) chicken intestine. The small and large intestines of ED18 chicken embryos were embedded in Tissue-Tek^®^ (Sakura Finetek, Alphen aan den Rijn, the Netherlands) and frozen using liquid nitrogen. Cryosections (4 µm) of the ED18 intestine were obtained using a microtome and placed on glass slides (Superfrost/plus, Germany). ED18 intestinal cryosections were first fixed with 4% PFA, permeabilized with Triton X-100 for 10 min at RT, and blocked in blocking buffer for 2 h. Intestinal sections were then incubated with primary antibodies as mentioned in Table 1 in humified chamber at 4 °C overnight. The next day, secondary staining was performed followed by nuclear staining with DAPI. In between the staining steps, intestinal sections were washed with washing buffer.

Intestinal tissues sections and monolayer cultures were imaged using Leica TCS SPE-II microscope (Leica, Amsterdam, The Netherlands) using 20×, 40×, 63×, or 100× ACS apo cs oil objective, and images were analyzed with FIJI software (NIH, version ImageJ 1.52r). The spheroid size were measured manually.

### Periodic acid-Schiff (PAS) staining

Mucus-producing goblet cells in monolayer culture were identified by Periodic acid-Schiff (PAS) staining method. Monolayer cultures were fixed with hemacolor solution-1 (Sigma-Aldrich) for 1 h and incubated with 0.5% PAS (Sigma-Aldrich) for 5 min at RT, followed by washing with distilled water three times and staining with Schiff’s reagent (Sigma-Aldrich) for 40 min. The samples were then counterstained with hematoxylin (Sigma-Aldrich) for 1 min at RT and mounted on FluorSave reagent with monolayer faced down on Polysine^®^ microscope slides. Glass slides were analyzed under Olympus BX41 microscope (Olympus, Leiderdorp, The Netherlands) using 20× and 40× plan phase objectives.

### Immunoblot analysis

Cell lysates were dissolved in RIPA Lysis and Extraction buffer (Thermo-Scientific, Landsmeer, The Netherlands, 89900) and Halt Phosphatase Inhibitor Cocktail (Thermo-Scientific, 78420) was added to the samples. Proteins (30 μg/well) were separated by SDS-PAGE using pre-cast gel 10% Tris/Gly (Bio-Rad, Lunteren, the Netherlands). After transfer to PVDF membrane (0.22 µm), blots were probed with anti-flag HRP 1:500 (Invitrogen), or β-actin (#4967) and visualized using horseradish peroxidase-labeled anti-rabbit antibodies and chemiluminescence on a Bio-rad ChemiDoc XRS + system. The experiments were repeated 3 times.

### Dot blot analysis

Supernatant from Wnt3 and RSPO1 producing Hek293t cells were collected and 5 µl of the supernatant was spotted on nitrocellulose membrane and dried for 30 min. Blocking of non-specific sites were done by incubating in 5% BSA in TBS-T for 1 h at RT. Next, anti-flag HRP (1:500) was added, for 60 min at RT and subsequently rinsed with TBS-T (5x) and visualized using chemiluminescence on a Bio-rad ChemiDoc XRS + system. The experiments were repeated three times.

### Real-time quantitative PCR

The gene expression level of various passages of 3D and 2D intestinal organoids was determined by Real-Time quantitative PCR (RT-qPCR). Two wells of 3D and 2D intestinal organoids culture were harvested from Matrigel using cold Advanced DMEM/F12 medium by vigorous pipetting and centrifuged at 295×*g*, 5 min at 4 °C. The pellet was lysed in RLT buffer (Qiagen, Venlo, the Netherlands) supplemented with β-mercaptoethanol (Sigma-Aldrich). Total RNA was extracted using RNeasy mini kit (Qiagen, 74106) according to manufacturer’s instructions and RNA was quantified using NanoDrop spectrophotometer (Isogen, de Meern, The Netherlands).

Reverse transcription of 0.5 µg of total RNA was done using iScript cDNA synthesis kit (Bio-Rad, 170-8891) as per manufacturer’s instructions and cDNA was diluted 1:1 ratio in milli-Q. RT-qPCR assays were performed using CFX384 real-time PCR detection system (Bio-Rad). Primer LGR5 (Forward: CCCACTGCTATCAGGACACTAAC; Reverse: GAGGCACCATTTAAAGTCAGAG) was used as a marker for stem cells. Normalization of LGR5 was done using GAPDH (Forward: GTGGTGCTAAGCGTGTTATC; Reverse: GCATGGACAGTGGTCATAAG) as the housekeeping gene. 5 µL of cDNA template was added per well consisting of 20 µL reaction mixture using 12.5 µL SYBR Green Supermix, 5.5 µL of distilled water, 1 µL (400 nM) of forward and reserve primers. PCR thermal cycle conditions used were as follows: an initial 5 min step at 95 °C, followed by 40 cycles of 92 °C for 10 s, 55 °C for 10 s, and 72 °C for 30 s. This was followed by cycle 3 consisting of 95 °C for 1 min, and 65 °C for 2 min.

### Microarray

The microarray was performed as described previously^23^. Briefly, One hundred nanogram of RNA was used for Whole Transcript cDNA synthesis (Affymetrix, inc., Santa Clara, USA). Hybridization, washing and scanning of Affymetrix GeneChip Mouse Gene 1.1 ST arrays was carried out according to standard Affymetrix protocols. All arrays of the small intestine were hybridized in one experiment. Arrays were normalized using the Robust Multiarray Average method^24,25^. Probe sets were assigned to the unique gene identifiers Entrez IDs. The probes on the Mouse Gene 1.1 ST arrays represent 21,213 Entrez IDs. Array data were analyzed using an in-house, on-line system^26^.

### Data analysis

Significant differences in the size of organoids when culturing with different supplements, and the mRNA levels of LGR5 between multiple passage numbers, were analyzed by the Kruskal–Wallis test, followed by the Dunn’s multiple comparisons test in Graphpad Prism (version 9.3.1, San Diego, CA, USA). A P-value < 0.05 as considered significant.

## Results

### Chicken-derived growth factors WNT3 and RSPO1 are preferred for the maintenance of chicken intestinal organoids

**Figure 2:**
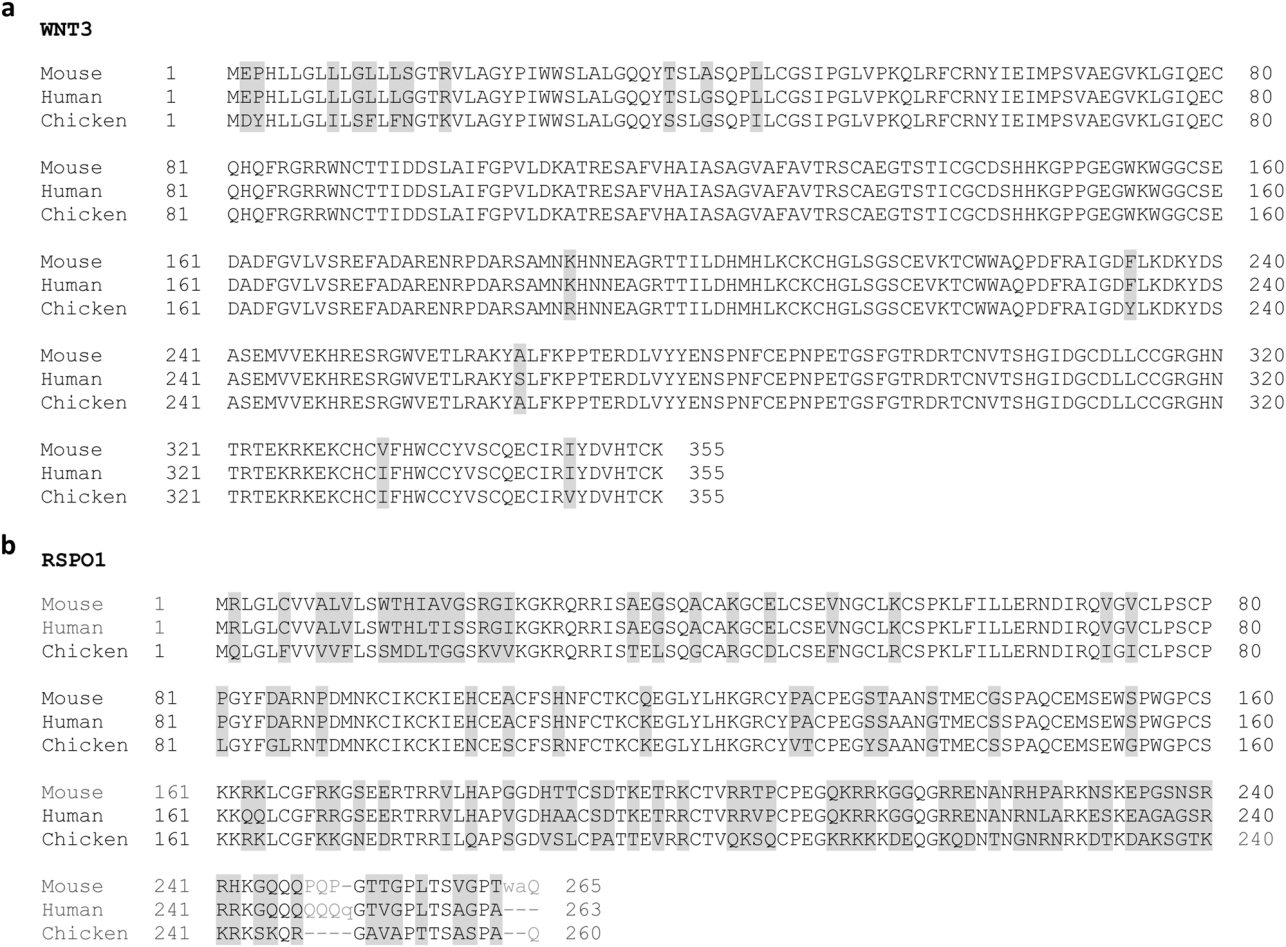
Sequence alignments of (**a**) WNT3 protein for mouse, human and chickens. Chicken vs mouse and chicken vs human are 96% identical (human vs mouse: 98%). (**b)** Alignment of RSPO1 for mouse, human and chicken. Chicken and mouse is 65% identical and chicken and human is 68% (human and mouse: 89%). Non-consensus amino acids (grey).

Alignments of WNT3 and RSPO1 between human, mouse, and chicken shows that chicken WNT3 is 96% identical to mouse and human Wnt3 (Fig. 2a). Moreover, chicken RSPO1 is only 65% identical to mouse Rspo1 and 68% identical to human RSPO1 (Fig. 2b). This indicates that in chickens, both growth factors are different from the mammalian orthologue, therefore, urging us to produce chicken-specific WNT3 and RSPO1.

**Figure 3:**
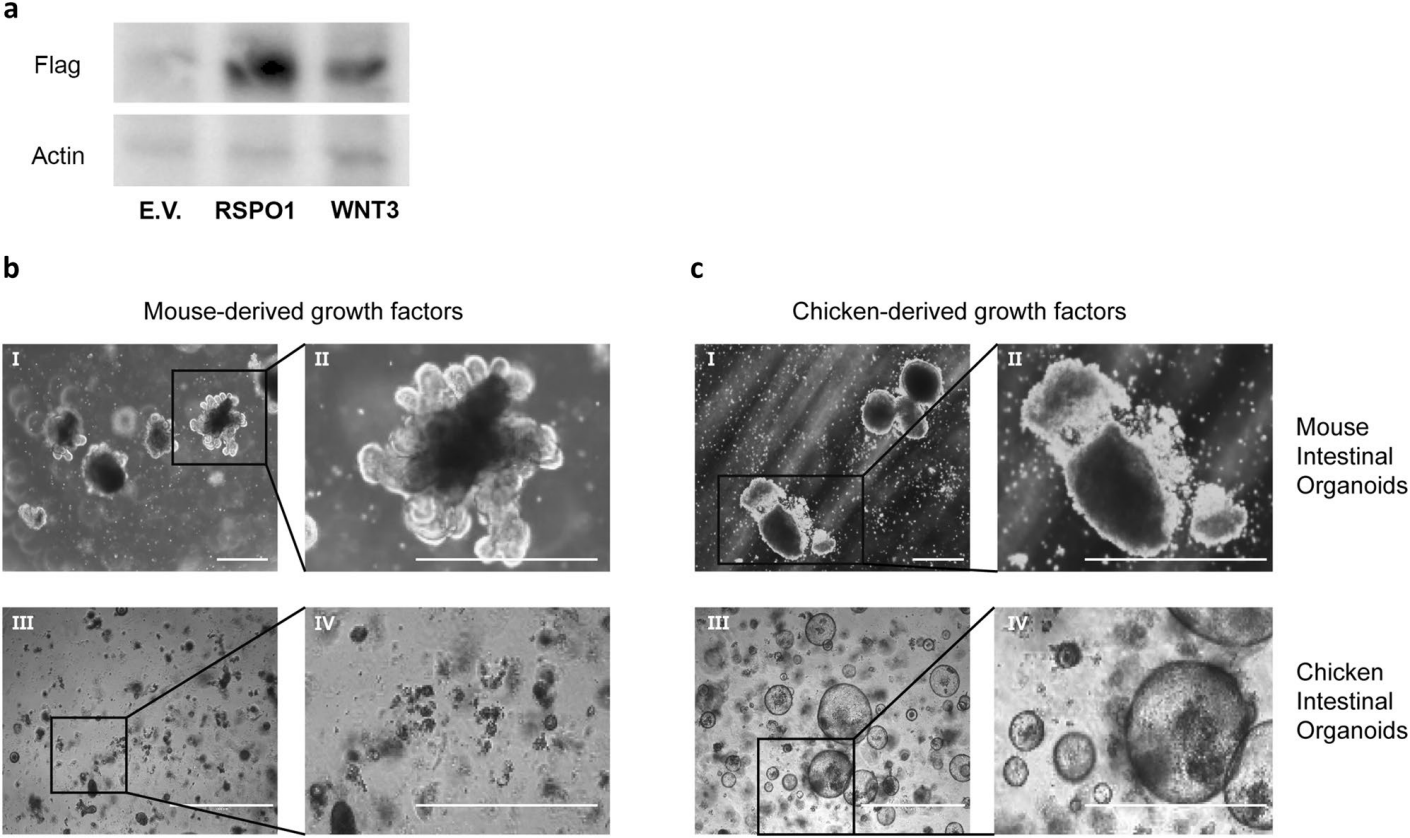
Growth factors RSPO1 and WNT3, from murine or chicken origin, are important for establishing intestinal organoid growth. **(a)** Immunoblot analysis of chicken RSPO1 and chicken WNT3 protein expression in transfected Hek293t cells. Actin was used as the protein loading control and anti-flag HRP was used to visualize RSPO1 and WNT3. Empty Vector (E.V.) is taken along as a control. **(b)** Brightfield images of mouse intestinal organoids **(I,II)** and chicken intestinal organoids **(III,IV)** at passage 2 (5 days after passage). Cultured in Matrigel with ICM, supplemented with Rspo1 and Wnt3 of murine origin,** (c)** or supplemented with chicken-derived RSPO1 and WNT3. Images and immunoblot are representative of three independent cultures. Scale bar 200 µm.

Hek293t cells expressing chicken WNT3 and RSPO1 were developed to obtain these growth factors for organoid cultures. Immunoblot analysis of a cell lysate of transfected cells showed a single protein of approximately 28 kD for both growth factors when stained with their cognate antibodies (Fig. 3a; full-length blot Fig. S1). Moreover, by making use of a dot blot, we showed that WNT3 and RSPO1 are successfully secreted in the supernatant (Fig. S2). The effect of chicken-derived WNT3 and RSPO1 were compared to the mouse-derived growth factors on cultures of chicken intestinal organoids and murine intestinal organoids (Fig. 3b,c). To this end, chicken intestinal organoids and murine intestinal organoids were cultured in ICM, supplemented with murine-derived Wnt3 and Rspo1 (MOCM) or with chicken-derived WNT3 and RSPO1. After 1 week, chicken intestinal organoids cultured in ICM supplemented with chicken-derived WNT3 and RSPO1 showed growth of spheroid-like structures, which increased in number (Fig. 3c (III, IV)), whereas only dark structures were observed when culturing chicken organoids in MOCM (Fig. 3b (III, IV)). When mouse organoids were cultured in MOCM, the growth and formation of lobular structures were observed (Fig. 3b (I, II)). Culturing mouse intestinal organoids in ICM supplemented with chicken-derived WNT3 and RSPO1 resulted in dark structures, without lobular structures (Fig. 3c (I, II)). Altogether, these data indicate that chicken organoids indeed require chicken-specific growth factors RSPO1 and WNT3 for optimal growth. Furthermore, we observed that, in contrast to mouse intestinal organoids, chicken intestinal organoids grow as spheroids rather than lobular structures (Fig. 3c (III, IV)).

### Defining optimal culture conditions for chicken intestinal organoids

**Figure 4:**
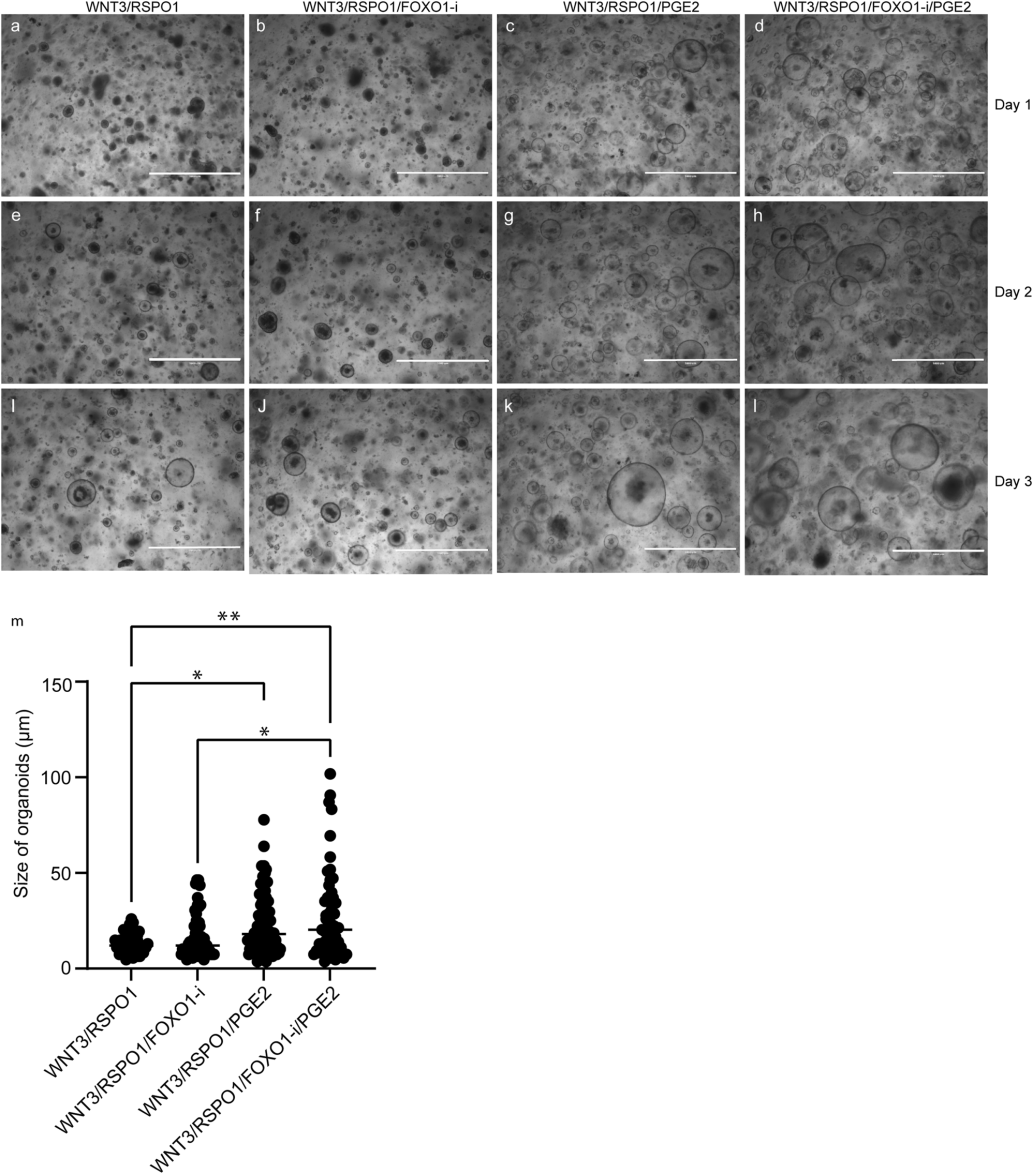
Supplementary compounds for organoid growth. Brightfield images of embryonic chicken organoid cultured in Matrigel with BCM of passage 1, 1,2 and 3 days after splitting. (**a,e,i)** with additional RSPO1 and WNT3 of chicken origin. (**b,f,j)** with additional RSPO1/WNT3 of chicken origin and 0.2 µM Forkhead box O1-inhibitor (FOXO1-i). (**c,g,k)** with additional RSPO1/WNT3 and 2.5 µg/ml prostaglandin E2 (PGE2) (**d,h,l)**. With all 4 supplemented growth factors (WNT3/RSPO1/PGE2/FoxO1-i). Images are representative of three independent cultures. Scale bar: 1000 µm. **(m)** Diameter of organoids when culturing with different supplements, were measured manually and plotted in Graphpad Prism. Four outliers were excluded after performing an outlier test in Graphpad Prism. Statistics were performed using a Kruskal–Wallis test followed by Dunn’s multiple comparisons test. *p < 0.05 **p < 0.01.

Next, we evaluated the effect of additional components, such as PGE_2_ and the FOXO1-inhibitor AS1842856 on the growth of chicken intestinal organoids. It is known from literature that PGE_2_ has a positive effect on the growth of chicken intestinal organoids and FOXO1 plays a role in stemness^21,27^. We therefore cultured freshly isolated embryonal chicken intestinal organoids in ICM with chicken-derived WNT3 and RSPO1 (Fig. 4a,e,i), either supplemented with the FOXO1-inhibitor AS1842856 at a concentration of 0.2 µM (Fig. 4b,f,j) or PGE_2_ at a concentration of 2.5 µg/ml (Fig. 4c,g,k) or both. The growth of organoids was monitored for 3 days. The addition of the FOXO1-inhibitor resulted in similar organoid growth and structures when compared to the ICM with chicken-derived WNT3 and RSPO1 cultures (Fig. 4m). Interestingly, supplementation of PGE_2_ resulted in more and larger spheroids (Fig. 4m). Moreover, when PGE_2_ was combined with the FOXO1-inhibitor, we observed an increase in spheroid diameter, compared to PGE_2_-only exposed cultures.

### The LGR5+ intestinal stem cells stay stable during multiple passages

**Figure 5:**
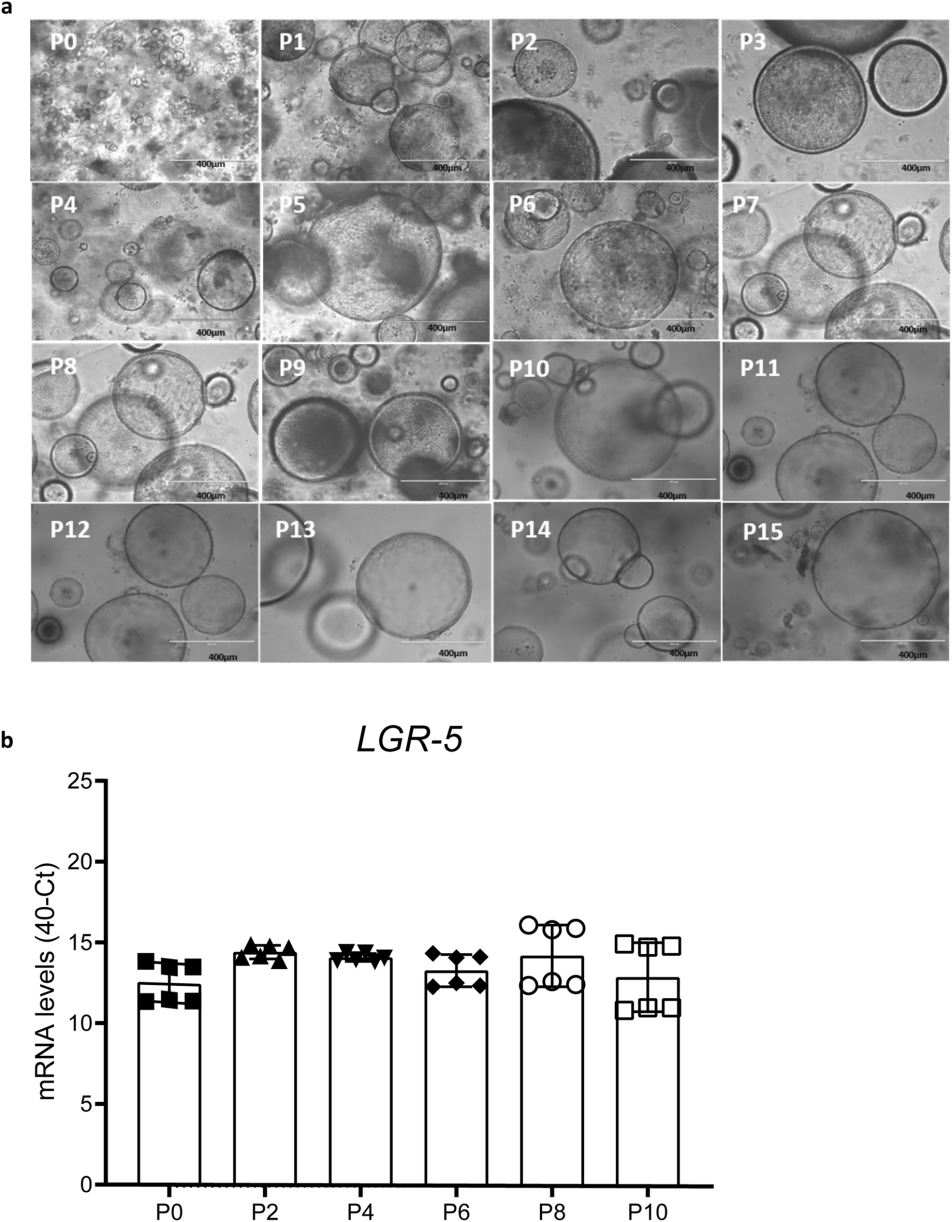
Longevity of the chicken intestinal organoids. **(a)** Brightfield images of chicken intestinal organoids cultured in chicken organoid culture medium. All pictures were taken 2 days after passaging. Scale bar: 400 µm. (**b)** mRNA levels of LGR5 in 3D embryonic chicken intestinal organoids, expressed as 40-Ct values with GAPDH as a reference gene. Two independent isolations were performed in technical triplicates. The error bars represent the standard deviation of the mean.

To our knowledge, there are no publications that show the same efficient growth of embryonal chicken intestinal organoids, compared to mammalian species, for longer than 21 days. Therefore, we tested the longevity of the embryonic chicken intestinal organoid cultures. Organoid cultures were passaged every 3–4 days and the growth in size and number of the organoid culture was monitored over time (Fig. 5a). Up until ten passages (approximately 5 weeks), we observed fast-growing spheroids, however, later cultures had reduced growth and after 15 passages this resulted in a low passage efficiency.

Organoid cultures are highly dependent on LGR5-expressing stem cells and thus the observed growth suggests that stem cells are present in these cultures (Fig. 5a). To confirm the presence of these stem cells, we analyzed the mRNA expression of the stem cell marker *LGR5* by RT-qPCR for the first ten passages and observed similar expression levels of *LGR5* in all the passage numbers tested (Fig. 5b). This suggests that the stem cell population stayed constant over time.

### Cellular diversity of chicken intestinal organoids is comparable to intestinal tissue of chicken

**Figure 6:**
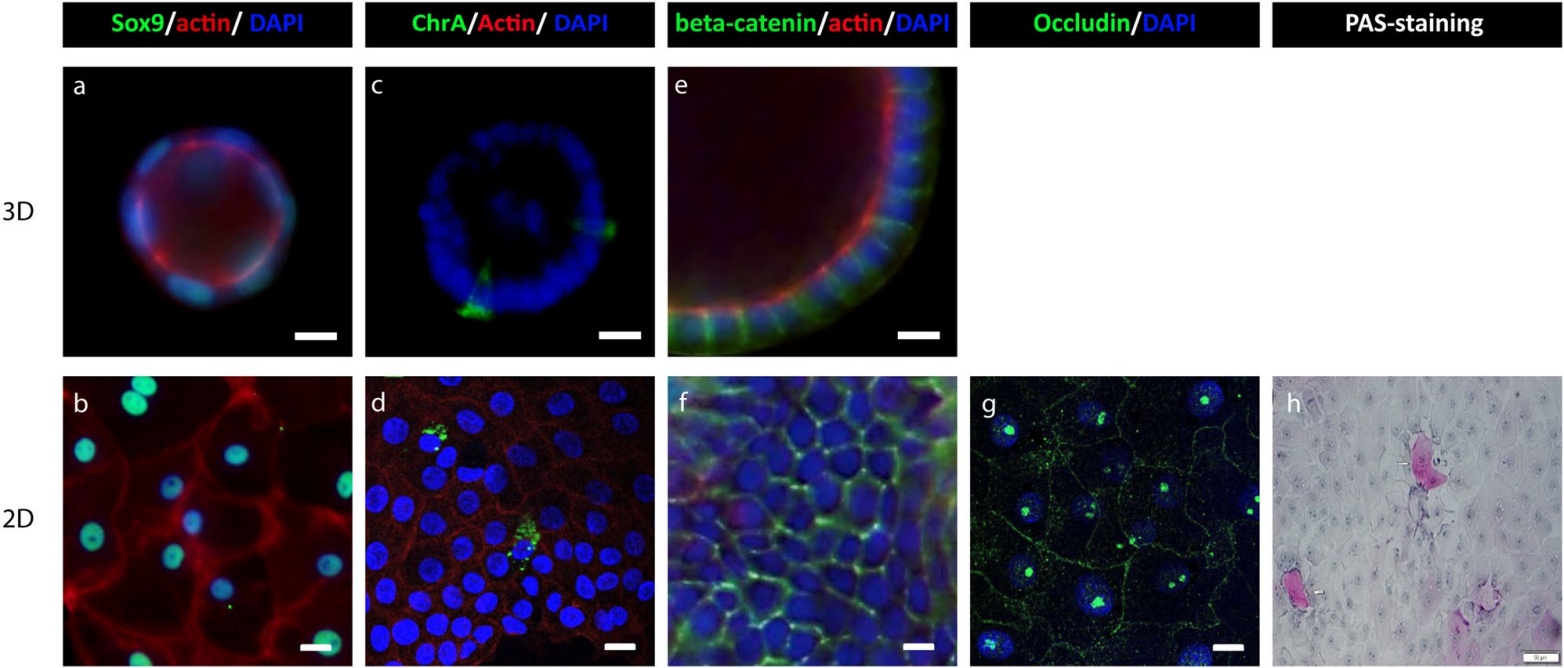
Identification of different cell structures in 3D chicken intestinal organoid and the monolayers. Immunohistochemistry images of 3D and 2D structures of chicken intestinal organoids, processed 2 days after seeding. **(a,b)** Progenitor cells are visualized with anti-Sox9. **(c,d)** Enteroendocrine cells are visualized by Chromogranin A. **(e–g)** Tight junctions are visualized with beta-catenin or occludin **(h)** Mucus-containing goblet cells are visualized using Periodic Acid Schiff (PAS)-positive cell staining of 2D chicken intestinal organoids. The actin filaments are visualized with rhodamine phalloidin (red), and nuclei are stained with DAPI (blue) Scale bar: 400 µm.

For mouse and human intestinal organoids, it has been nicely established that the LGR5+ stem cells can differentiate into other intestinal cells, such as goblet cells, enteroendocrine cells, and enterocytes^28,29^. To investigate the cellular diversity of the embryonal chicken intestinal organoids, we performed 3D cultures as well as 2D cultures. The 2D cultures allow us to study the differentiation of stem cells into enterocytes and functional enteroendocrine cells, since enterocytes do not grow well in Matrigel, which is also supported by mRNA expression of specific enterocyte genes in 2D and 3D murine intestinal organoids (Fig. S3)^11,23^. Subsequently, immunohistochemistry and PAS cell staining were performed on these 3D and 2D cultures (Fig. 6). First, to confirm that the antibodies are cross-reactive with chickens, embryonic intestinal tissue was used as a control and showed that the antibodies are functional in chicken tissue (Fig. S4). To identify stem cells and transit-amplifying cells, both 2D and 3D cultures were stained for SOX9. This resulted in a nuclear staining for both 3D and 2D (Fig. 6a,b, Fig. S5). Anti-Chromogranin-A was used as a marker for enteroendocrine cells and allowed us to detect this specific cell type in 3D as well as in 2D culture (Fig. 6c,d). Goblet cells can produce mucins that can be stained using PAS reagents^30^. We observed a few PAS-positive cells in 2D chicken organoids, indicating that our cultures contain goblet cells (Fig. 6h). Subsequently, the presence of epithelial barrier proteins, such as CTNNB1 (β-catenin) and OCLN (Occludin), was determined by immunohistochemistry. Β-catenin is present at cell–cell junctions in 3D as well as in 2D. Occludin is shown to be present in the 2D chicken intestinal organoids (Fig. 6e,f,g). Overall, this data shows that the different cell-types that have been described for mammalian intestinal organoids are also present in embryonal chicken intestinal organoids when cultured in COCM, indicating that these chicken organoids maintained their capacity to differentiate into the different intestinal specific cell types.

## Discussion

In this study, we aimed to optimize the culture of chicken intestinal organoids and to extend their longevity (i.e. number of passages) compared to what has been reported so far^12–14,16^. We show that by using growth factors of chicken origin, instead of using the commercially available mammalian growth factors WNT3 and RSPO1, cultures could be passaged up to 15 times. Moreover, we show that the growth and longevity of these cultures can be enhanced by addition of PGE_2_ and by inhibiting FOXO1. We also demonstrate that these organoids can be grown in 2D as well as 3D and have the capacity to differentiate into intestinal specific cell types, such as goblets, endocrine cells and enterocytes, which offers a broad application of chicken organoids.

It is known that the WNT, NOTCH, and BMP signalling pathways play a pivotal role in the proliferation and differentiation of intestinal stem cells^10,31–33^. Wnt3 and Rspo1 are used in mammalian intestinal organoid cultures^10,28^. Apart from the addition of Wnt3 and Rspo1, the small molecules CHIR99021 and valproic acid support the maintenance of self-renewal of intestinal stem cells and growth of organoids in mice by enhancing the expression of WNT-target genes^34^, and also improved growth of chicken intestinal organoids^17^. However, for chicken intestinal organoids, culturing with mammalian Wnt3a and Rspo1 did not result in successful long-term organoid cultures (Fig. 3)^12,13,16,19^. Powell et al., did show multiple passages, however, they observed that growth of chicken organoids is far less efficient than it is observed for mammalian organoids^15^. In addition, Zhao et al. have shown that chicken intestinal organoids were not viable for longer than 3 weeks when using the mammalian Wnt3a and Rspo1^35^. Therefore, we investigated the effect of the species-specific WNT3 and RSPO1 and observed that chicken intestinal organoids benefit from the medium supplemented with chicken-derived WNT3 and RSPO1. Our results indicate that COCM allows growth of chicken intestinal organoids which can be maintained for more than 15 passages. The benefit of culturing for multiple passages is that we can produce faster and more robust outcomes and it is more sustainable compared to short-term cultures, concerning lab animals.

To further optimize the culture conditions, additional effects of PGE_2_ and inhibition of FOXO1 were studied. PGE_2_ evidently supports the growth of chicken intestinal organoids, compared to ICM with Chicken-derived WNT3 and RSPO1 (Fig. 4). These findings extend those of Pierzchalska and colleagues, confirming a more defined and more rigid form of chicken intestinal organoids^14,19^. Although Pierzchalska already showed spheroid-forming chicken intestinal organoids, they were not capable of long-term cultures. By adding the FOXO1-inhibitor, we found an increase in number and size of intestinal organoids, suggesting that by inhibiting FOXO1, we can enhance the growth of organoids. This is in line with the research of Choi et al., where it was demonstrated that there is negative crosstalk between LGR5+ stem cells and FOXO1, in which FOXO1 inhibits the self-renewal capacity of gastric cancer cells^20^. So, inhibiting FOXO1 would contribute to the activation of self-renewal.

Our embryonal chicken intestinal organoids grow as spheroids instead of the lobular structures, which are mostly observed in human and mouse intestinal organoids^10,28^. However, in contrast to most mammalian organoid papers^10^, we do not isolate crypts from adult chickens, but rather from embryonal chickens. When we isolate crypts from adult chickens, they also show spheroid structures, however, it was not possible to split these organoids beyond passage 1 (data not shown). A recent paper from Mustata and colleagues showed that embryonal crypts from mouse intestines are also growing as spheroids, similar to the structures that we have observed in our cultures, suggesting that the spheroid-like growth might be due to their embryonic nature. Moreover, they showed that mouse embryonal spheroids turn into adult lobular organoids within 10 passages. If embryonal organoids are destined to become adults in vitro, then this might explain why we lose growth after 15 passages, as we already indicated that the current COCM is not suitable for growth of adult chicken organoids.

A unique feature of organoids, compared to most cell lines, is their capacity to form different cell types which all have their specific function in the intestine, such as barrier function, hormone secretion and absorption and metabolism of nutrients. By staining for specific markers we show that most of these cells are present, especially in 2D, thus reflecting the same plasticity of these long-term cultures as has been published for mammalian organoids^36^. However, we also stained for progenitors by using SOX9 as a marker. It was against our expectation that there were also many SOX9 positive cells in 2D cultures, where there is limited matrigel present that is normally required to maintain stemness and thus proliferation. A possible explanation for this might be the presence of CHIR99021 and VA that enhance the WNT-pathway.

Organoids can be an important tool for the reduction of animal experiments and allow for efficient testing of multiple potentially health-promoting feed additives or pharmaceuticals. The ability to grow chicken intestinal organoids for more passages reduces the need for test animals and increases the possibility of more mechanistic research of pathways involved in intestinal health, metabolism, absorption, barrier function and host-microbe interactions. To study these interactions more in-depth, organoid cultures can be expanded by co-culturing them with, for instance, immune cells and fibroblasts or microorganisms.

## Supplementary Information

Supplementary Information.

## Data Availability

The datasets used and/or analyzed during the current study available from the corresponding author on reasonable request.
