# Supplementary material for: Chicken-derived RSPO1 and WNT3 contribute to maintaining longevity of chicken intestinal organoid cultures
Source: Sci Rep. 2022 Jun 22;12:10563. doi: 10.1038/s41598-022-14875-7 (PMC9217957; doi:10.1038/s41598-022-14875-7)
Supplement: Supplementary file 1 — Supplementary Information. [file 41598_2022_14875_MOESM1_ESM.pdf]

Supplementary files

Table 1. Medium and reagents

Overview of all the reagents in Basic culture medium, Intermediate culture medium, Mouse organoid culture medium and Chicken organoid culture medium.

**Reagent**	**Basic culture medium (BCM)**	**Intermediate culture medium (ICM)**	**Mouse organoid culture medium (MOCM)**	**Chicken organoid culture medium (COCM)**
DMEM/F12	100%	100%	100%	100%
10% FCS	10%	10%	10%	10%
Glutamax	1%	1%	1%	1%
sodium pyruvate	1mM	1mM	1mM	1mM
Non-Essential Amino Acids	1mM	1mM	1mM	1mM
Penicillin-streptomycin	10,000 U/mL	10,000 U/mL	10,000 U/mL	10,000 U/mL
CHIRR99021		4.3 µM	4.3 µM	4.3 µM
Y27632		10 µM	10 µM	10 µM
Valproic acid		0.78 mM	0.78 mM	0.78 mM
DMH-1		1.3 µM	1.3 µM	1.3 µM
Mouse-specific Wnt3a			1:10	
Mouse-specific Rspo1			1:10	
FOXO1-inhibitor AS1842856				0.2 µM
Prostaglandin E2				7.09 µM
Chicken-specific WNT3				1:5
Chicken-specific RSPO1				1:5

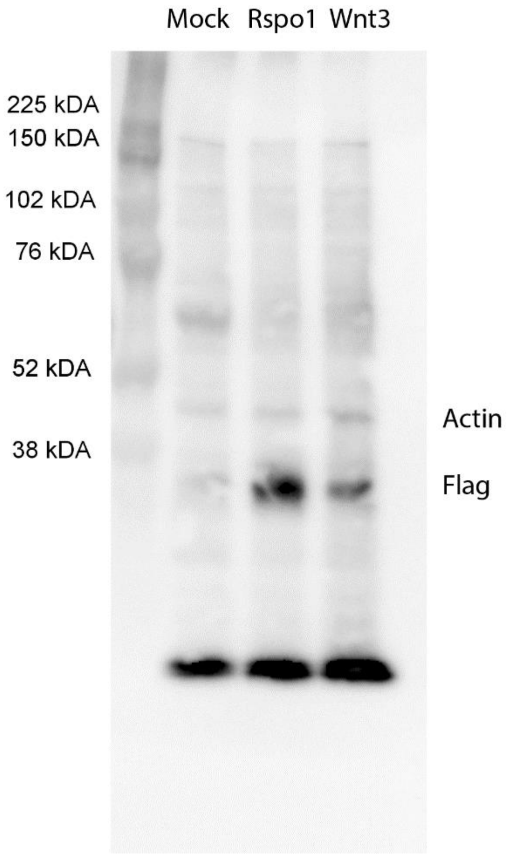

Supplementary figure 1. Immunoblot of chicken RSPO1 and chicken WNT3 protein expression in transfected HEK293T cells.

Actin was used as the protein loading control and anti-flag HRP was used to visualize RSPO1 and WNT3.

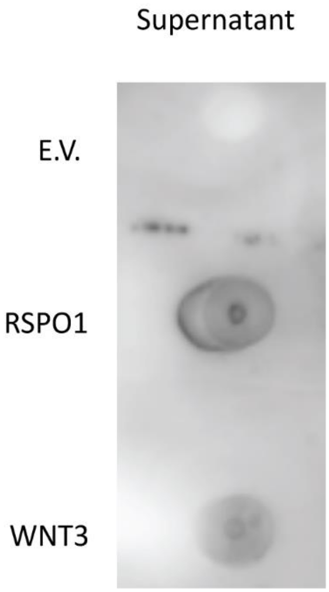

Supplementary figure 2. Dot-blot of chicken RSPO1 and chicken WNT3 protein expression in supernatant of transfected Hek293t cells.

Empty vector (E.V.), chicken RSPO1 and chicken WNT3 are detected by anti-flag-HRP.

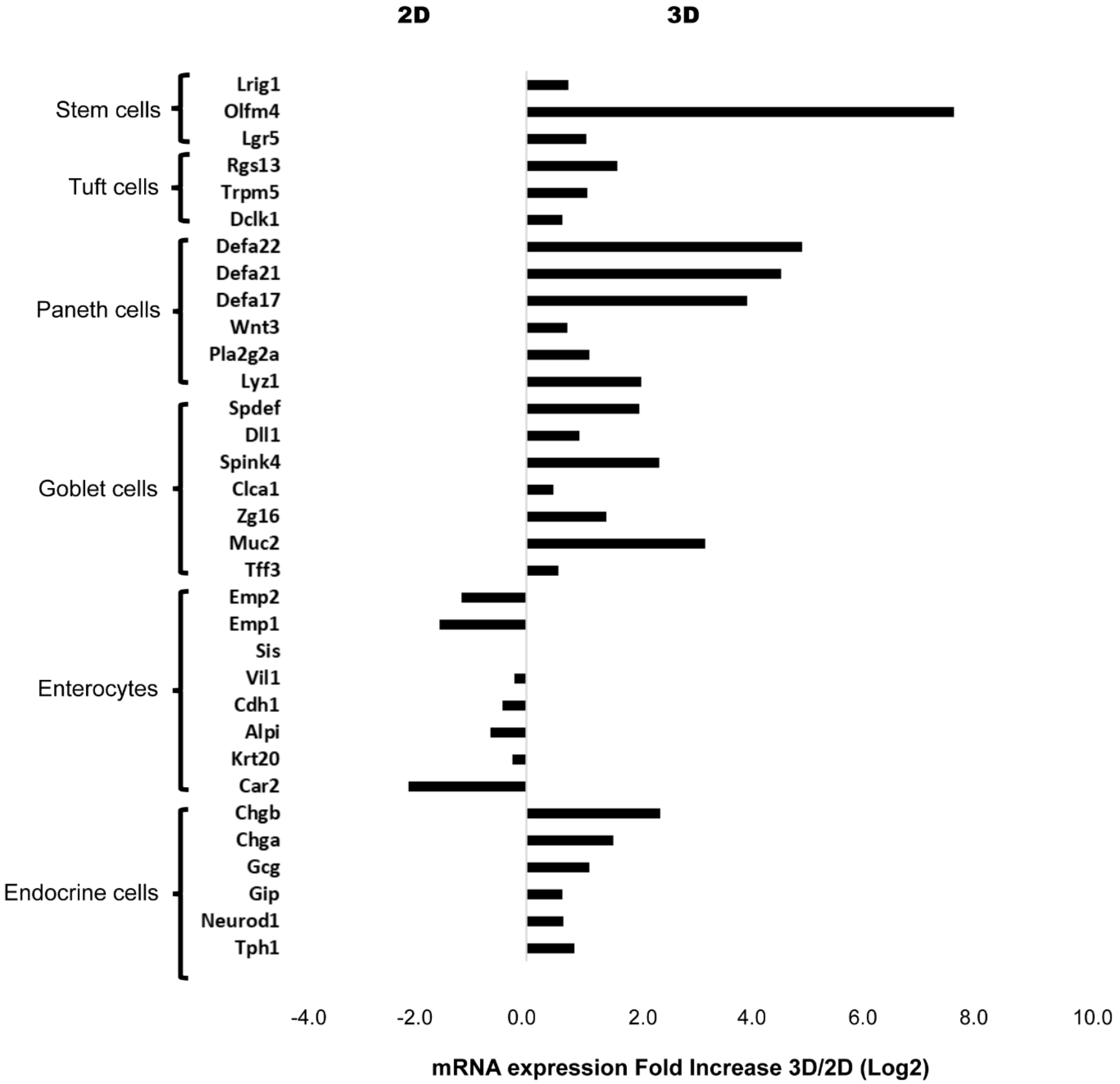

Supplementary figure 3. mRNA expression of cell-specific genes expressed in 2D and 3D mouse intestinal organoids.

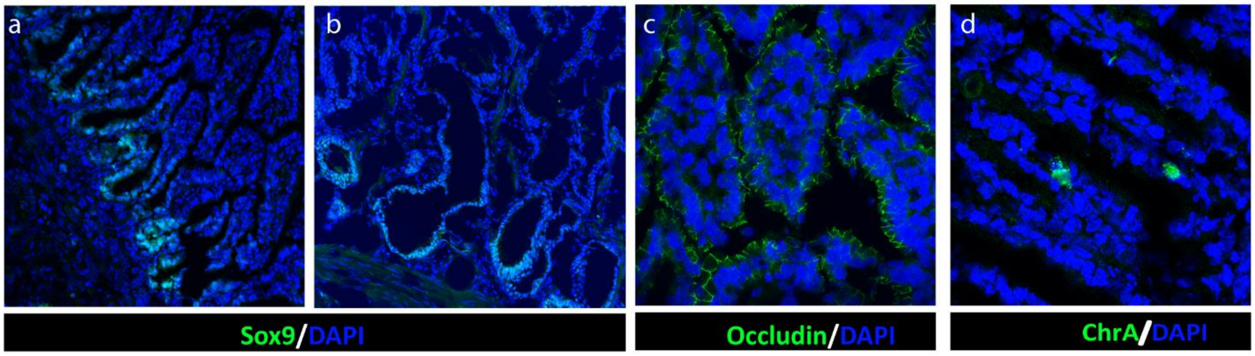

Supplementary figure 4. Confirming functionality of antibodies in chicken tissue.

Immunohistochemistry images of embryonic intestinal tissues stained with antibodies mentioned in Table 1**. (a)** Progenitor cells are visualized with anti-Sox9 in embryonic tissue (magnification 20 x) (**b)** and in adult intestinal tissue **(c)** Tight junctions are visualized with anti-occludin (magnification 63 x) (**d)** Enteroendocrine cells are visualized by anti-chromogranin A. Nuclei are stained with DAPI (blue) (magnification 63x)

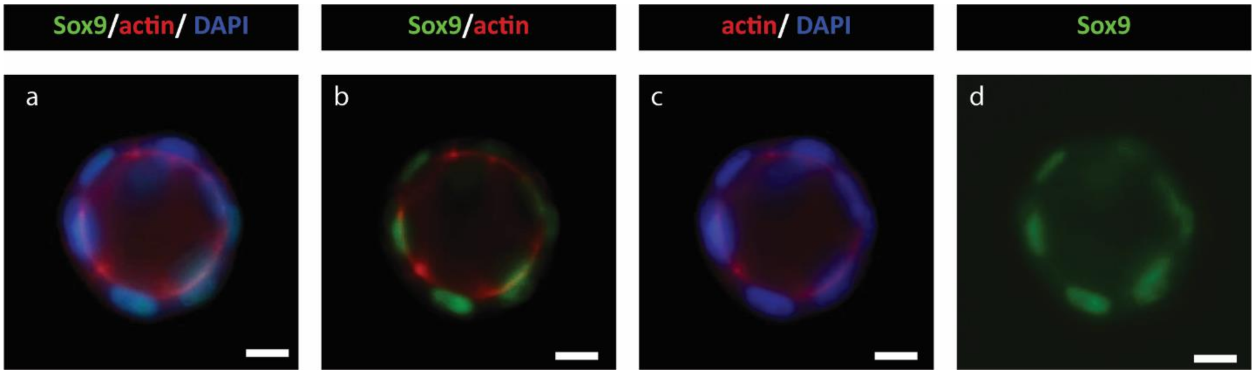

Supplementary figure 5. Immunohistochemistry images of SOX9 in 3D structures of chicken intestinal organoids Processed 2 days after seeding. Progenitor cells are visualized with anti-Sox9. **a.** Sox9/actin and DAPI staining **b.** Sox9/actin staining **c.** actin/ DAPI staining **d.** Sox9 staining. Scale bar: 400 µm.
